# Single-Cell RNA Sequencing Reveals Lactylation Modifications in Neuroblastoma and the Construction of a Prognostic Model

**DOI:** 10.3390/molecules31132280

**Published:** 2026-06-29

**Authors:** Wuhe Jike, Ke Tian, Junming Zhu, Kutluk Kasim, Xiao Zhi, Lufeng Cheng, Xuejun Xiao

**Affiliations:** 1 Department of Pharmacology, College of Pharmacy, Xinjiang Medical University, No. 567 Shangde North Road, Shuimogou District, Urumqi 830017, China; jkwh323@163.com (W.J.); 17799753259@163.com (K.T.); 18197570299@163.com (J.Z.); 17799318365@163.com (K.K.); 2 School of Medicine, Shanghai Jiao Tong University, No. 800 Dong Chuan Road, Shanghai 200240, China; zhixiao@sjtu.edu.cn; 3 Xinjiang Key Laboratory of Biopharmaceuticals and Medical Devices, No. 567 Shangde North Road, Shuimogou District, Urumqi 830017, China; 4 Institute of Materia Medica, Xinjiang Medical University, No. 567 Shangde North Road, Shuimogou District, Urumqi 830017, China

**Keywords:** neuroblastoma, lactylation, prognostic model, single-cell RNA sequencing, risk stratification, molecular markers

## Abstract

Lactylation, a recently identified post-translational modification, has been associated with multiple cancer types, including neuroblastoma (NB). The present study aimed to investigate the prognostic significance of lactylation-related genes and to develop a prognostic model to enhance patient risk stratification and guide targeted therapy for NB. In the present bioinformatics study, single-cell RNA sequencing data (GSE137804) were analyzed to quantify lactylation activity in NB cells using the AddModuleScore algorithm based on 371 lactylation-related genes. A total of 142 differentially expressed lactylation-related genes (DELGs) were identified between high- and low-lactylation tumor cells, and these genes were mainly enriched in cell cycle-related pathways. A 14-gene lactylation-related prognostic model was then constructed using the identified DELGs in a training cohort (GSE49710, *n* = 349) via Cox and LASSO regression, and validated in internal (GSE49710, *n* = 149) and external (E-MTAB-8248, *n* = 223) cohorts. The model effectively stratified patients into high- and low-risk groups with significantly different overall survival (OS) outcomes, and its robust predictive performance was confirmed across both validation cohorts. The present study reveals the significant prognostic role of lactylation in NB, and the 14-gene model serves as a novel molecular tool for risk stratification and provides a reference for developing targeted therapeutic strategies for NB.

## 1. Introduction

Neuroblastoma (NB) is a common and aggressive pediatric solid tumor with high heterogeneity and poor prognosis, accounting for 8–10% of pediatric malignancies and 15% of cancer-related deaths in children [1,2]. Despite significant advances in multimodal therapy—including high-dose chemotherapy, autologous stem cell transplantation, and anti-GD2 immunotherapy—the 5-year overall survival (OS) of high-risk NB patients remains below 50% [3,4]. Importantly, recent randomized controlled trials have demonstrated that merely intensifying or extending conventional induction chemotherapy fails to improve event-free survival while increasing toxicity, underscoring an urgent need to move beyond traditional cytotoxic regimens and develop novel prognostic and therapeutic strategies [5,6]. Clinical presentation and prognosis of NB patients vary greatly, and conventional prognostic markers (age at diagnosis, clinical stage, *MYCN* amplification, etc.) offer limited predictive value, particularly in capturing the biological heterogeneity that drives treatment failure [4,7]. Recent efforts leveraging machine learning and deep learning have improved NB risk stratification using multiphase CT imaging or alternative transcriptomic signatures; however, these approaches either rely on indirect imaging phenotypes or focus on cellular pathways distinct from emerging metabolic-epigenetic drivers [8,9]. Moreover, the systemic immune landscape of pediatric solid tumors, including NB, is highly variable and remains poorly characterized, with implications for both prognosis and the development of effective immunotherapies [10]. Thus, novel molecular prognostic tools and therapeutic targets are urgently needed to improve NB patient outcomes.

Lactylation is a recently identified post-translational modification (PTM) wherein a lactyl group derived from lactate is covalently attached to lysine residues of proteins, a process primarily catalyzed by lactate dehydrogenase [11,12]. This modification has emerged as a crucial regulator of tumor biology, influencing cellular energy metabolism, gene transcription, cell cycle progression, and immune microenvironment remodeling [12]. Consequently, lactylation plays a central role in tumor proliferation, invasion, and therapy resistance [12]. For example, histone lactylation promotes tumorigenesis by activating oncogene expression or facilitating immune evasion in various cancers [13,14,15]. Importantly, a recent study in NB demonstrated that *DNAJC12* downregulation enhances glycolysis and lactic acid production, leading to increased histone H4 lysine 5 lactylation (H4K5la) and activation of invasion- and metastasis-associated transcriptional programs, suggesting a potential role of lactylation in NB progression [16]. Recent high-quality studies and reviews have further indicated that histone and non-histone lactylation, including L-/D-/S-lactylation, participate broadly in oncogenic transcription, metabolic adaptation, tumor immune regulation, and therapeutic vulnerability [17]. In addition, lactylation has been implicated in cancer responses to radiotherapy and immunotherapy [18], hypoxia-induced metabolic-epigenetic adaptation [19], glycolysis–histone lactylation-positive feedback during tumorigenesis [20], and macrophage-mediated immunosuppression within the tumor microenvironment [14,21]. However, research focusing on lactylation in NB is limited, and the expression patterns, prognostic significance, and regulatory mechanisms of lactylation-related genes in NB are yet to be fully elucidated.

**Figure 1:**
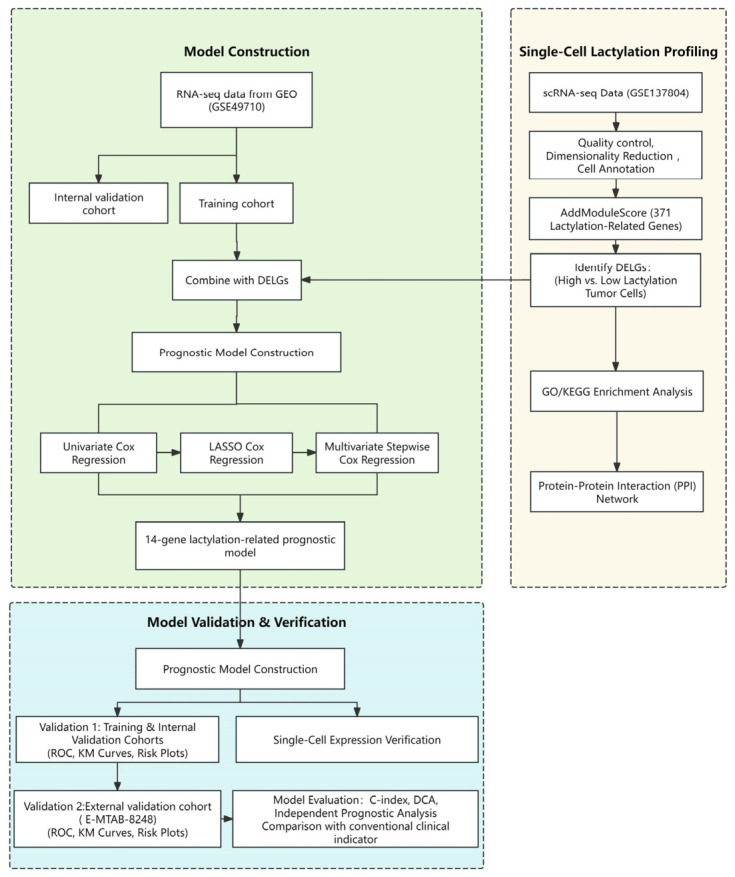
Technical Route.

Recently, several prognostic tools have been developed for NB, including image-based deep learning models, population-based clinical nomograms, and transcriptomic signatures [21,22]. Notably, Zhu et al. recently reported a seven-gene lactylation-related prognostic model for NB with a focus on the PI3K/AKT-metabolic axis and *KLHL32*-mediated immune modulation. While their study provided valuable experimental insights, their model emphasized metabolic–immune crosstalk rather than tumor-cell-intrinsic proliferative drivers. A prognostic model that captures the transcriptional footprints of tumor-cell-specific lactylation in cell cycle regulation and chromosomal instability remains lacking. Furthermore, no lactylation-based prognostic stratification tool has yet been established specifically from NB tumor-cell-derived differentially expressed genes at single-cell resolution. We therefore hypothesized that lactylation activity is dysregulated—and potentially elevated—in NB tumor cells, and that lactylation-related gene expression signatures may serve as reliable prognostic biomarkers. To test this hypothesis, we integrated single-cell RNA sequencing (scRNA-seq) and bulk transcriptomic data to: (1) characterize lactylation activity across NB cell populations at single-cell resolution; (2) identify differentially expressed lactylation-related genes (DELGs) and their associated functional pathways; and (3) construct and validate a lactylation-related prognostic model for NB risk stratification. The overall workflow of this study is depicted in Figure 1.

## 2. Results

### 2.1. scRNA-Seq Data Processing and Cell Annotation

**Figure 2:**
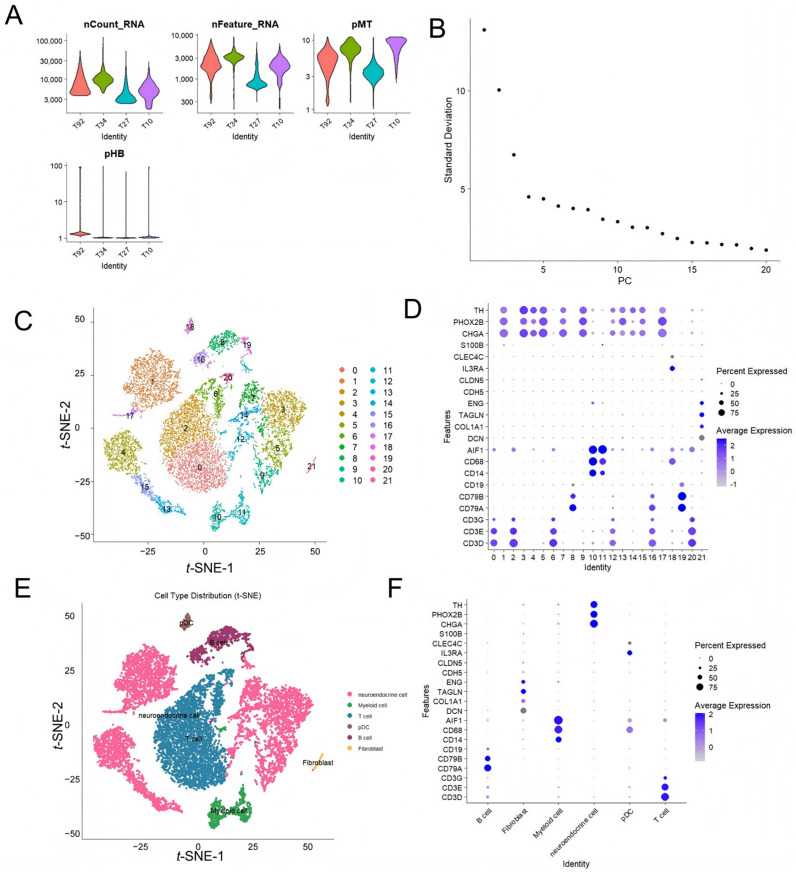

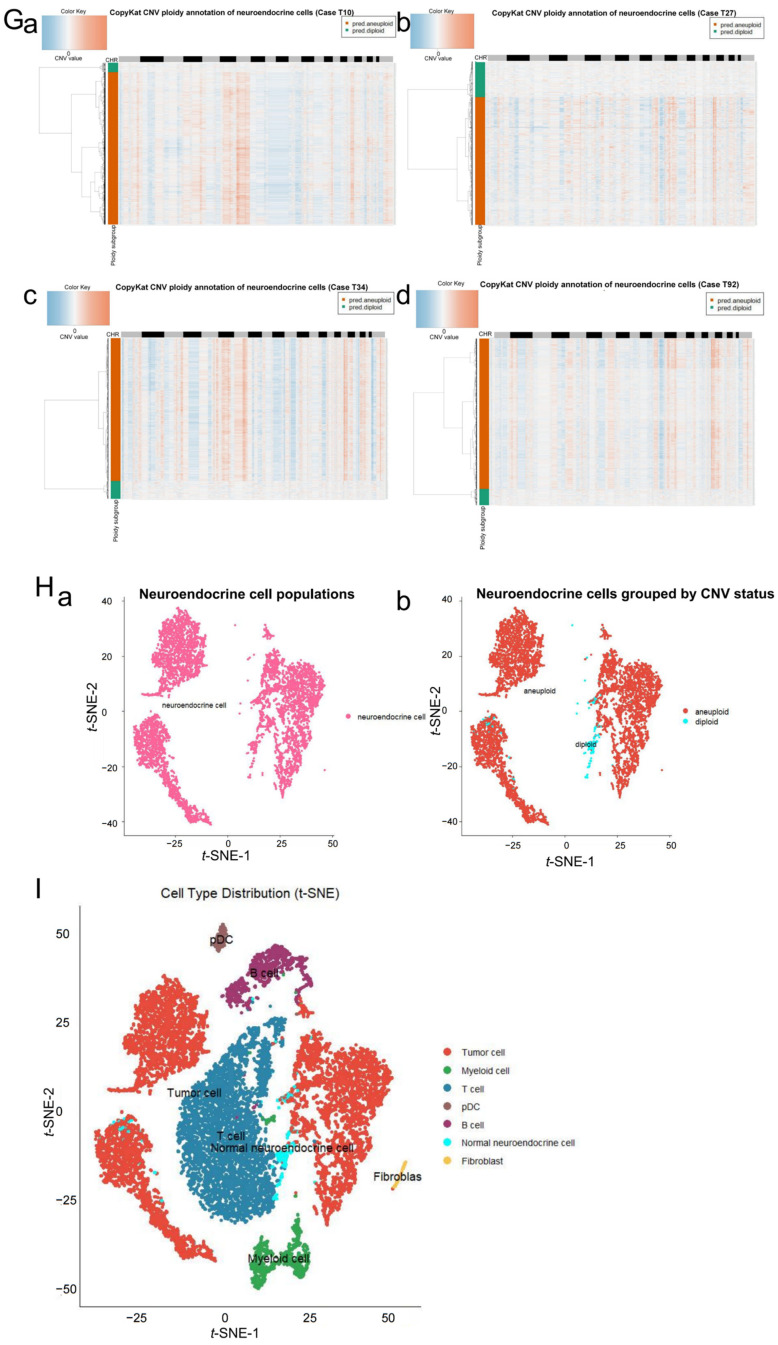
scRNA-seq data processing, quality control, and cell type annotation. (**A**) Violin plots display key quality control metrics, including the number of genes, total UMI counts, and the percentage of mitochondrial genes per cell. (**B**) An elbow plot visualizes the standard deviations of the first 20 principal components from the PCA. (**C**) Cell clusters identified by the Louvain algorithm are visualized in a *t*-SNE plot. (**D**) A dot plot shows the average expression and percentage of canonical marker genes in each cluster. (**E**) Cell types are preliminarily annotated based on marker gene expression and visualized in a *t*-SNE plot. This neuroendocrine cell population comprises both tumor and normal neuroendocrine subpopulations, which are further stratified by CNV status in (**G**,**H**). (**F**) Expression verification of key marker genes for major cell types is presented in a dot plot. (**G**) CopyKAT CNV ploidy profiling of neuroendocrine cells from four representative patient samples. Subpanels (**a**–**d**) correspond to cases T10, T27, T34 and T92, respectively. Orange indicates predicted aneuploid (tumor) cells, and green denotes predicted diploid (normal) cells. (**H**) *t*-SNE visualization of neuroendocrine cell subgroups classified by CopyKAT CNV results. (**a**) Unseparated full neuroendocrine cell population; (**b**) Cells stratified by ploidy status: red = aneuploid tumor cells, cyan = diploid normal cells. (**I**) The final annotation of seven distinct cell types is visualized in a *t*-SNE plot. Normal and tumor neuroendocrine cells were separated from the same cluster via CopyKAT based on CNVs; their partial overlap represents early malignant transitional cells.

After quality control (QC), 15,281 high-quality cells were retained from the initial 1,631,502 cells (Figure 2A). Principal component analysis (PCA) showed that the top 10 principal components accounted for 76.71% of the cumulative variance (Figure 2B), and 21 cell clusters were identified by the Louvain algorithm (Figure 2C). Based on marker gene expression, the clusters were initially annotated into six cell types (Figure 2D,E). Key marker gene expression was verified and is shown in a dot plot (Figure 2F). Furthermore, subsequent copy number variation (CNV) analysis distinguished tumor cells from normal neuroendocrine cells (Figure 2G,H), resulting in seven major cell types in NB tissues: tumor cells (51.14%), normal neuroendocrine cells (1.57%), myeloid cells (5.65%), T cells (32.87%), B cells (6.78%), pDCs (1.17%), and fibroblasts (0.82%) (Figure 2I).

### 2.2. Global Upregulation of Lactylation Levels in NB Tumor Cells

**Figure 3:**
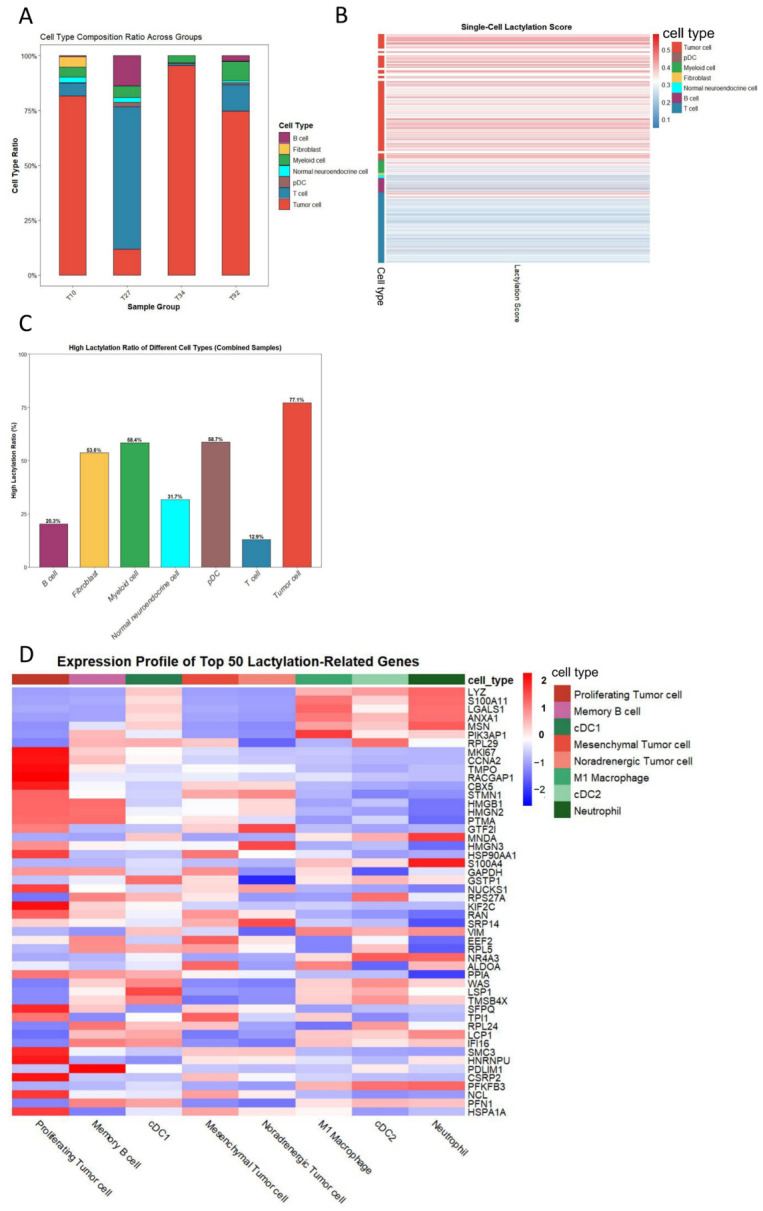
Analysis of lactylation activity across cell types and populations. (**A**) A stacked bar chart displays the proportional composition of major cell types across the four samples. (**B**) A heatmap displays the average lactylation scores per cell type. (**C**) The proportion of cells with high lactylation activity in each annotated cell type is shown in a bar chart. (**D**) Expression profile of the top 50 lactylation-related genes.

The cell type composition across the four samples was heterogeneous, as shown in the stacked proportion plot (Figure 3A). As illustrated in the heatmap (Figure 3B), lactylation levels were consistently higher in tumor cells than in other cell types. The bar chart (Figure 3C) further quantified the elevated lactylation activity in tumor cells: 77.1% of tumor cells exhibited high lactylation activity (above the global median), whereas only 31.7% of normal neuroendocrine cells and less than 60% of non-tumor cells (including myeloid cells, T cells, and B cells) fell into the high-lactylation category. The results collectively indicated that lactylation activity was significantly elevated in NB tumor cells.

To further resolve cellular heterogeneity, 18 refined cell subtypes were annotated: Proliferating Tumor cells, Memory B cells, cDC1s, Mesenchymal Tumor cells, Noradrenergic Tumor cells, M1 Macrophages, cDC2s, Neutrophils, pDCs, Fibroblasts, γδ T cells, Treg cells, Normal neuroendocrine cells, CD8+ T cells, M2 Macrophages, CD4+ T cells, Activated B cells, and Plasma cells. The eight subtypes listed above exhibited lactylation levels above the global median threshold and were therefore defined as high-lactylation cell types. The expression heatmap of lactylation-related genes across the high-lactylation subtypes was shown in Figure 3D. Proliferating tumor cells specifically expressed high levels of cell cycle regulators (e.g., *MKI67*, *CCNA2*). In contrast, immune-related genes were predominantly expressed in non-tumor cells. The data provided a single-cell-resolution expression atlas linking lactylation-related genes to functionally distinct NB cell subpopulations.

### 2.3. DELGs Are Enriched in Cell Cycle Pathways and Core Gene Identification

**Figure 4:**
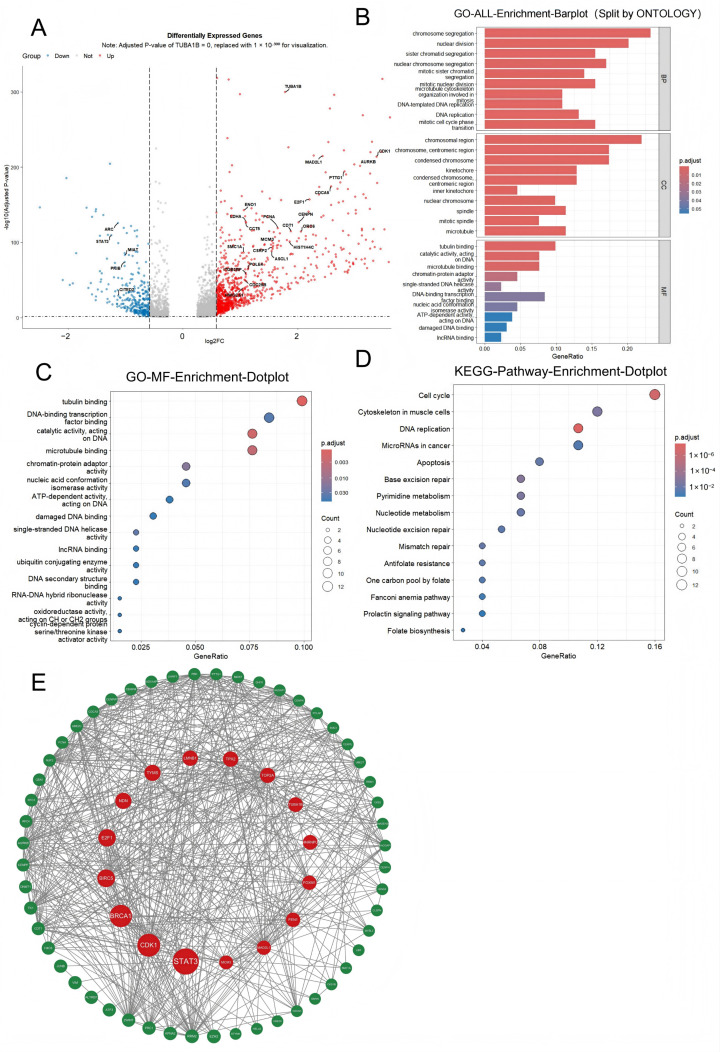
Functional enrichment and protein–protein interaction (PPI) network analysis of differentially expressed lactylation-related genes (DELGs). (**A**) Differential analysis between the high- and low-lactylation groups is presented. (**B**) Significantly enriched Gene Ontology (GO) terms are displayed in a bar chart. (**C**) Key GO enrichment results are also visualized in a bubble chart. (**D**) Significantly enriched KEGG pathways are presented in a bubble chart. (**E**) A PPI network is constructed based on the DELGs.

Differential expression analysis was performed between tumor cells from the high- and low-lactylation groups to identify genes whose expression was associated with lactylation status. In this analysis, the log2 fold change (log2FC) served as a quantitative metric for assessing the strength of association between lactylation status and gene expression. This rationale is supported by the established role of histone lactylation as an epigenetic mark that directly activates gene transcription [8,10], as well as by accumulating evidence that lactylation can modulate the function of transcription factors and chromatin regulators, thereby indirectly reshaping global transcriptional programs [11,12,13,14,15,16]. Thus, genes exhibiting substantial log2FC between lactylation groups are considered candidate downstream effectors potentially influenced by lactylation-mediated regulation, providing a transcriptomic basis for prioritizing targets for subsequent functional investigation. A total of 142 differentially expressed lactylation-related genes (DELGs) were identified (Table S1). The results are visualized in a volcano plot (Figure 4A). Among the DELGs, 113 genes were upregulated and 29 genes were downregulated in the high-lactylation group. As shown in the volcano plot, the average log2FC ranged from 1.00 to 3.60 for upregulated genes and from −2.49 to −1.00 for downregulated genes. All identified DELGs were statistically significant (*p <* 0.05). The 142 DELGs thus served as core targets for further investigation.

GO enrichment analysis showed that DELGs were significantly enriched in DNA-binding activity and catalytic activity acting on DNA at the molecular function level (adjusted *p* = 0.0012–0.00199); chromosome segregation, mitotic cell cycle phase transition, DNA-templated DNA replication, and positive regulation of the cell cycle at the biological process level (adjusted *p* = 4.95 × 10^−8^–1.41 × 10^−18^); and chromosomal region, condensed chromosome, kinetochore, and microtubule at the cellular component level (adjusted *p* = 2.28 × 10^−5^–2.44 × 10^−19^) (Figure 4B,C).

KEGG pathway analysis revealed DELGs were mainly enriched in the cell cycle (adjusted *p* = 2 × 10^−7^) and DNA replication (adjusted *p* = 3 × 10^−8^) pathways (Figure 4D). Genes involved in the cell cycle pathway included *CDK1*, *AURKB*, *PTTG1*, *CDCA5*, *MAD2L1*, *E2F1*, *ORC6*, *CDT1*, *MCM3*, *PCNA*, *CDC25B*, and *SMC1A*, while those related to the DNA replication pathway included *FEN1*, *MCM3*, *PCNA*, *RFC3*, *POLE4*, *RFC4*, *RNASEH2A*, and *RNASEH2B*.

Collectively, the findings indicated that DELGs were involved in the malignant progression of NB, which might be mediated through the modulation of critical biological processes such as the cell cycle and DNA replication. PPI network construction identified 101 DELGs with interactions (Figure 4E), and 16 core genes were identified based on the top quartile of betweenness centrality (BC) values (*STAT3*, *CDK1*, *BRCA1*, *BIRC5*, *E2F1*, etc.), which were key regulatory nodes of lactylation-related molecular networks in NB (Table S2).

### 2.4. Establishment of the 14-Gene Lactylation-Related Prognostic Model

**Figure 5:**
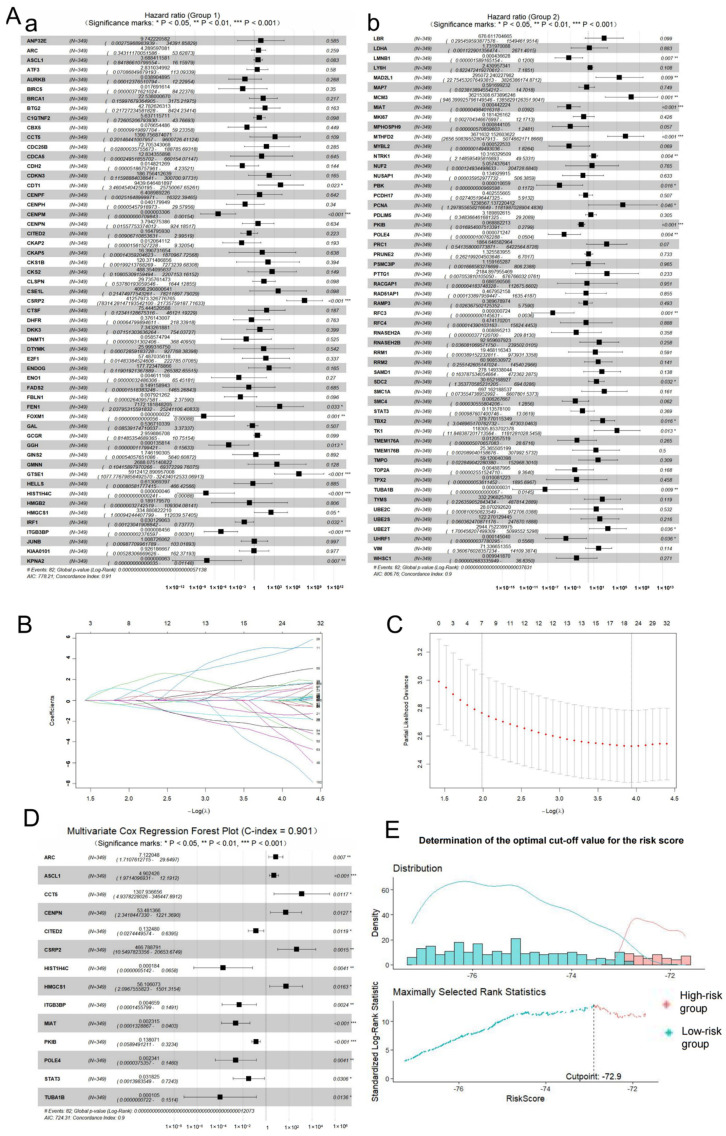
Construction of a lactylation-related prognostic risk model. (**A**) Univariate Cox regression analysis for candidate lactylation-related genes. Subpanels (**a**,**b**) separately display hazard ratio results of all genes due to the large number of candidate genes; genes significantly correlated with patient prognosis were screened at the threshold of *p* < 0.05. (**B**) The LASSO coefficient profiles of all candidate genes are shown. (**C**) The selection of the optimal penalization coefficient (λ) via 10-fold cross-validation in the LASSO regression model is displayed. (**D**) A forest plot displays the multivariate Cox regression results for the 14-gene lactylation-related prognostic model. (**E**) Determination of the optimal cut-off value for the risk score in the training cohort.

Univariate Cox analysis identified 109 DELGs significantly associated with neuroblastoma patient prognosis (*p* < 0.05), including 86 risk genes and 23 protective genes (Figure 5A). LASSO Cox regression with 10-fold cross-validation further screened 20 genes with non-zero coefficients at the optimal λ value: *ARC*, *ASCL1*, *CCT5*, *CENPN*, *CITED2*, *CSRP2*, *FEN1*, *HIST1H4C*, *HMGCS1*, *IRF1*, *ITGB3BP*, *LY6H*, *MIAT*, *MTHFD2*, *PKIB*, *POLE4*, *STAT3*, *TBX2*, *TUBA1B*, and *VIM* (Figure 5B,C).

Subsequent bidirectional stepwise regression ultimately identified 14 lactylation-related genes with independent prognostic value. Based on the identified genes, a neuroblastoma prognostic risk model was established (Figure 5D). The risk score was calculated as follows:

$$\begin{array}{l} \mathrm{Risk}\;\mathrm{Score}\;=\;(1.963\;\times ARC)\;+\;(1.590\;\times ASCL1)\;+\;(7.176\;\times CCT5)\\ +\;(3.979\;\times CENPN)\;+\;(-2.202\;\times CITED2)\;+\;(6.146\;\times CSRP2)\\ +\;(-8.601\;\times HIST1H4C)\;+\;(4.027\;\times HMGCS1)\;+\;(-5.369\;\times ITGB3BP)\\ +\;(-6.068\;\times MIAT)\;+\;(-1.980\;\times PKIB)\;+\;(-6.057\;\times POLE4)\\ +\;\left(-3.448\;\times STAT3\right)\;+\;\left(-9.166\;\times TUBA1B\right) \end{array}$$

In the formula, *ARC*, *ASCL1*, *CCT5*, *CENPN*, *CITED2*, *CSRP2*, *HIST1H4C*, *HMGCS1*, *ITGB3BP*, *MIAT*, *PKIB*, *POLE4*, *STAT3*, and *TUBA1B* represent the ${log}_{2}(x+1)$-transformed expression levels of the respective genes. Using the Log-rank test, the optimal cut-off value for the risk score in the training cohort was determined to be −72.9 (Figure 5E). The optimal cut-off value was used to stratify patients into high- and low-risk groups for subsequent survival analysis and model validation, which may serve as a reference for future clinical applications.

### 2.5. Evaluation of the Prognostic Model

#### 2.5.1. Overall Performance

Key metrics indicated excellent overall model performance. The concordance index (C-index) was 0.901, demonstrating a strong ability to discriminate patient survival risk. The global likelihood ratio test for the multivariate Cox model yielded a χ^2^ of 217.86 (*p* = 1.207 × 10^−48^), confirming the model’s high significance. An Akaike Information Criterion (AIC) value of 724.31 indicated a favorable balance between model fit and complexity.

#### 2.5.2. Validation Results of the Prognostic Model

**Figure 6:**
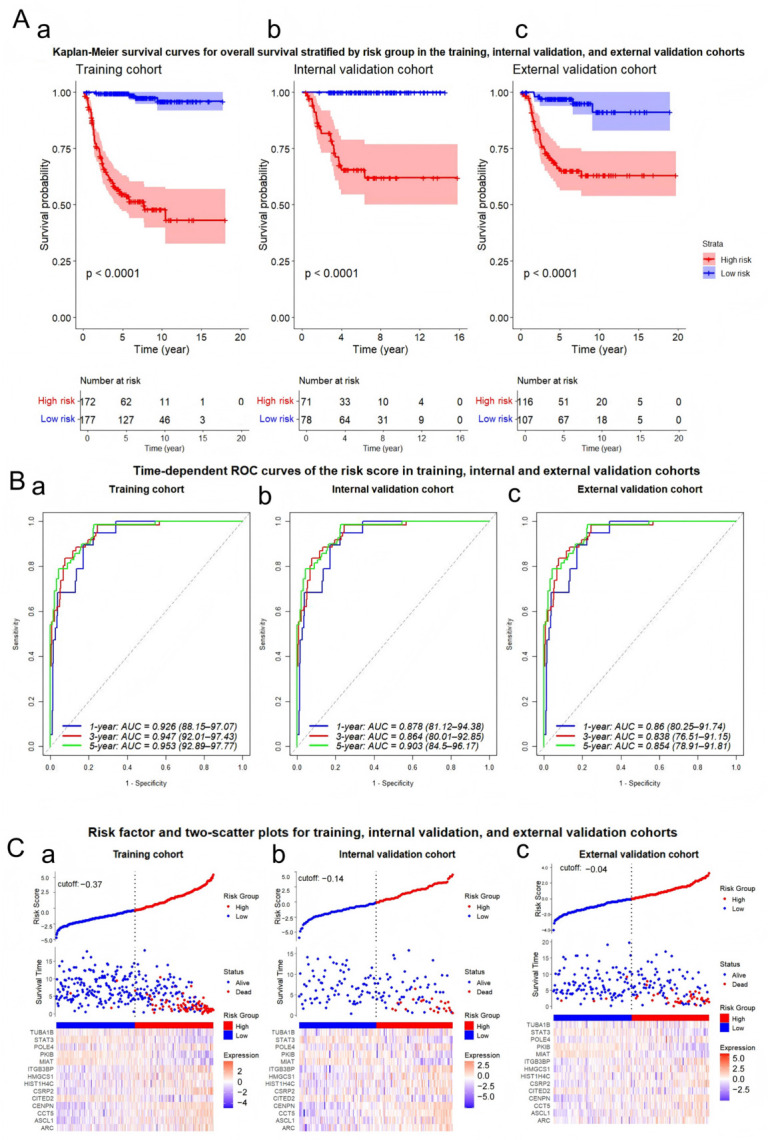
Validation of the lactylation-related prognostic risk model. (**A**) Kaplan–Meier survival curves comparing overall survival between the high- and low-risk groups: (**a**) training cohort, (**b**) internal validation cohort, (**c**) external validation cohort. (**B**) Time-dependent ROC curves evaluating the predictive performance of the risk model: (**a**) training cohort, (**b**) internal validation cohort, (**c**) external validation cohort. (**C**) Distribution of risk scores, patient survival status, and expression of the 14 signature genes across the three cohorts: (**a**) training cohort, (**b**) internal validation cohort, and (**c**) external validation cohort.

The prognostic performance of the 14-gene lactylation-related risk model was rigorously evaluated across the training (*n* = 349), internal validation (*n* = 149), and external validation (*n* = 223) cohorts. In all three cohorts, the model robustly stratified patients into high- and low-risk groups with significantly distinct overall survival outcomes (log-rank *p* < 0.0001 for all; Figure 6A).

Time-dependent ROC analysis demonstrated the model’s excellent and stable predictive accuracy. The AUC values for 1-, 3-, and 5-year OS were 0.947, 0.948, and 0.953, respectively, in the training cohort (Figure 6B(a)). The high predictive values were maintained in the internal validation cohort (3-year AUC = 0.864, 4-year AUC = 0.904, 5-year AUC = 0.903; Figure 6B(b)) and, importantly, in the independent external validation cohort (3-year AUC = 0.838, 4-year AUC = 0.845, 5-year AUC = 0.854; Figure 6B(c)), confirming the model’s generalizability.

Consistent with the risk score distribution, the heatmap analysis revealed a clear dichotomous expression pattern of the 14 core genes. In the high-risk group across all cohorts, risk-associated genes (e.g., *CCT5*, *CSRP2*) were predominantly upregulated, whereas protective genes (e.g., *TUBA1B*, *HIST1H4C*) showed higher expression in the low-risk group (Figure 6C). The dichotomous expression pattern underscored the biological relevance of the model in reflecting the underlying lactylation-related tumor biology.

#### 2.5.3. Comparison Results Between the Risk Score Model and Traditional Indicators

**Figure 7:**
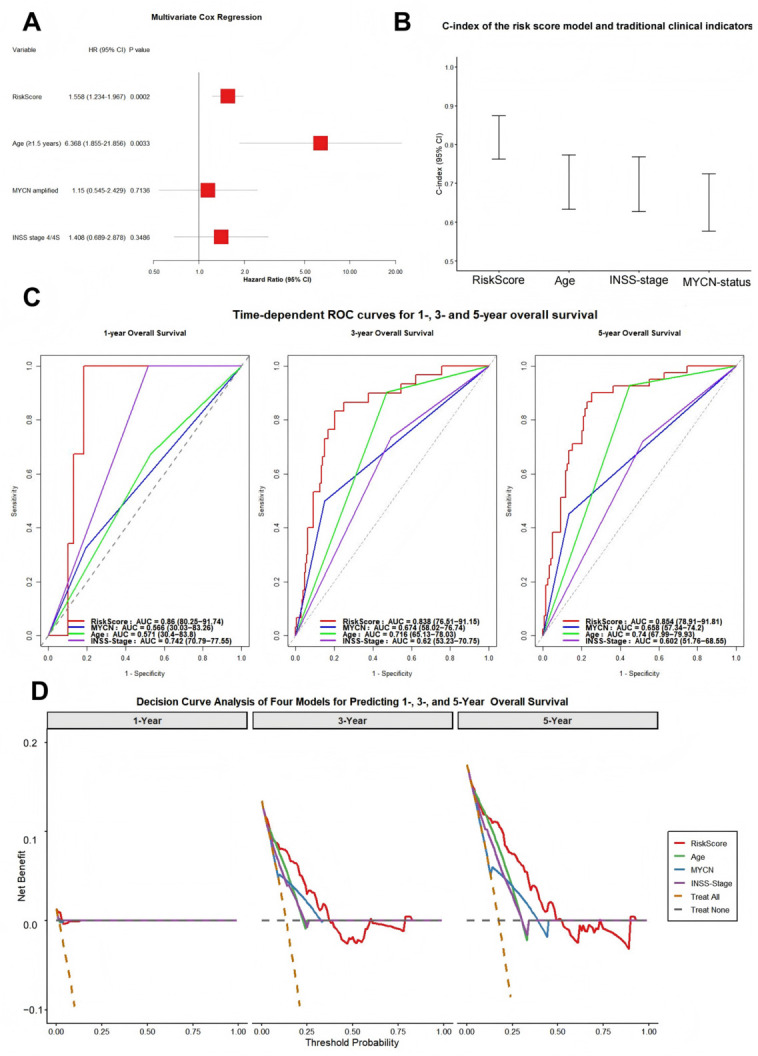
Performance comparison of the risk score model with traditional clinical indicators. (**A**) Multivariate Cox regression forest plot showing the hazard ratios (HRs) and the associated *p*-values of the risk score and conventional clinical factors for overall survival. The risk score was significantly associated with prognosis (HR = 1.558, 95% CI: 1.234–1.967, *p =* 0.0002). (**B**) Concordance index (C-index) comparison showing the superior discriminative ability of the risk score model. (**C**) Time-dependent ROC curves comparing the predictive accuracy of the risk score and traditional indicators for 1-, 3-, and 5-year overall survival. The risk score outperformed all clinical factors at all evaluated time points. The red curve corresponds to the risk score model. (**D**) Decision curve analysis demonstrating the higher clinical net benefit of the risk score model across a wide range of threshold probabilities. The red line represents the net benefit curve of the risk score model.

In the external validation cohort (E-MTAB-8248), multivariate Cox regression adjusting for age, *MYCN* status, and INSS stage confirmed that the 14-gene risk score was an independent prognostic factor for overall survival (HR = 1.558, 95% CI 1.234–1.967, *p =* 0.0002). Age ≥ 1.5 years was also significantly associated with worse survival (HR = 6.368, 95% CI 1.855–21.856, *p =* 0.0033). Neither *MYCN* amplification nor INSS stage 4/4S reached statistical significance in the cohort (*p >* 0.05), possibly because the risk score captured overlapping biological information related to tumor progression (Figure 7A).

The C-index analysis showed that the risk score model (C-index = 0.818, 95% CI 0.763–0.874) exhibited superior discriminative ability compared with age (0.703, 95% CI 0.633–0.773), INSS stage (0.698, 95% CI 0.628–0.768), and *MYCN* status (0.651, 95% CI 0.577–0.725) (Figure 7B).

In the external validation cohort (E-MTAB-8248), the time-dependent ROC analysis was performed to compare the predictive accuracy of the 14-gene risk score with established clinical factors (*MYCN* amplification, age ≥ 1.5 years, and INSS stage 4/4S) for 1-, 3-, and 5-year overall survival. The risk score consistently achieved the highest AUC values: 0.860 (95% CI 0.803–0.917) at 1 year, 0.838 (95% CI 0.765–0.911) at 3 years, and 0.854 (95% CI 0.789–0.918) at 5 years. The AUC values of the risk score were markedly higher than those of *MYCN* (AUCs ranging from 0.566 to 0.674), age (0.571 to 0.740), and INSS stage (0.602 to 0.742). The results demonstrated the superior and stable discriminatory power of the lactylation-related risk score over conventional prognostic markers (Figure 7C).

DCA showed that the risk score model provided higher net benefit than *MYCN* status, age, and INSS stage across a wide range of threshold probabilities for predicting 1-, 3-, and 5-year OS. The effective threshold range for the risk score expanded from 0–0.03 for 1-year survival to 0–0.37 for 3-year and 0–0.49 for 5-year survival, indicating broad clinical applicability (Figure 7D).

### 2.6. Single-Cell Expression Verification of the 14-Gene Prognostic Model

**Figure 8:**
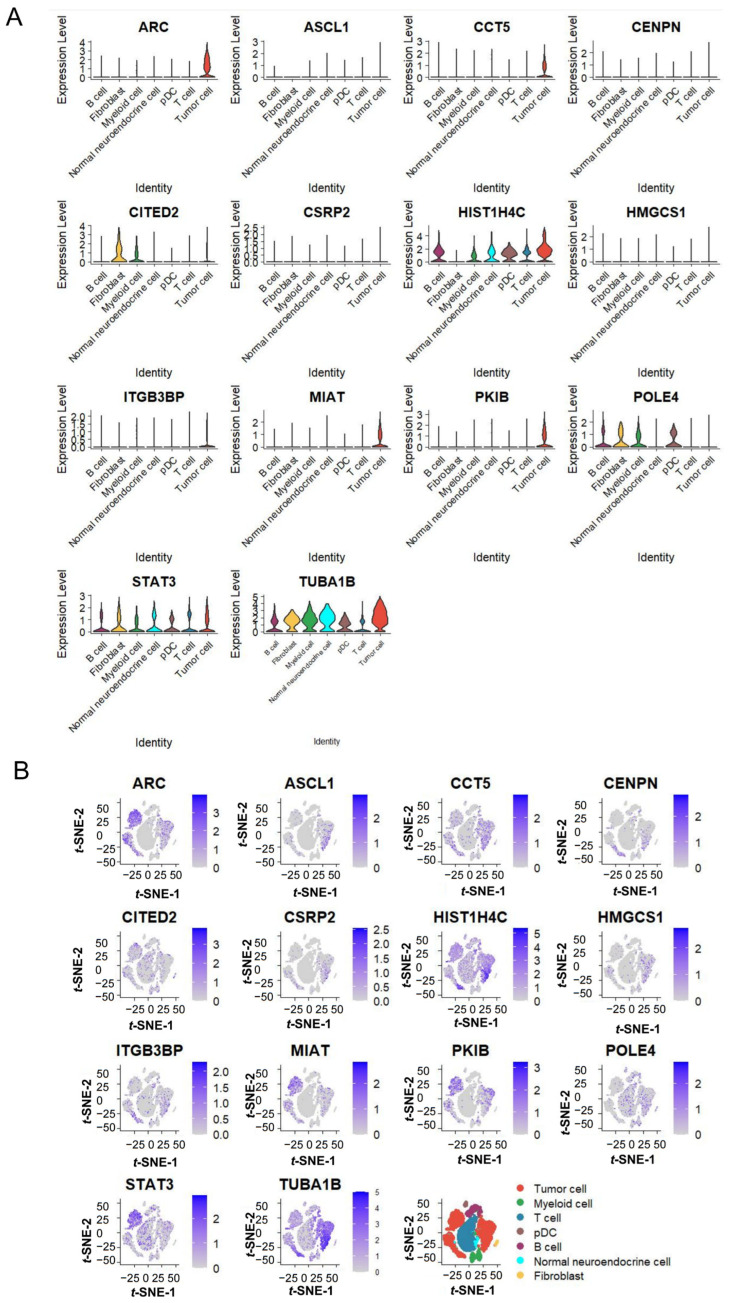
Single-cell expression landscape of the 14-gene prognostic model in neuroblastoma using the GSE137804 dataset. (**A**) Violin plots showing the expression distribution of each signature gene across different cell types (B cell, Fibroblast, Myeloid cell, Normal neuroendocrine cell, pDC, T cell, and Tumor cell). (**B**) *t*-SNE plots visualizing the spatial expression pattern of each gene at the single-cell level, with color intensity indicating the normalized expression level. The cell type annotation is shown in the legend. Notably, most signature genes exhibited preferential expression in tumor cells.

To further validate the biological relevance of the 14-gene prognostic model, we explored their expression patterns at single-cell resolution using the GSE137804 dataset. Violin plots (Figure 8A) showed that most risk genes, including *ARC*, *CCT5*, and *CENPN*, were specifically and highly expressed in tumor cells, whereas their expression in other cell populations (e.g., immune cells, stromal cells) was negligible. In contrast, protective genes such as *TUBA1B*, *HIST1H4C*, and *STAT3* were broadly expressed across multiple cell types, with the highest expression levels observed in tumor cells (Figure 8B).

## 3. Discussion

The extreme heterogeneity of NB is a major challenge for prognostic evaluation and treatment, and precision prognostic tools based on molecular features are crucial for improving patient outcomes. The present study integrated scRNA-seq and bulk transcriptomic data to systematically explore the role of lactylation in NB, constructed a 14-gene lactylation-related prognostic model with high predictive performance, and elucidated the underlying molecular mechanism, providing a new theoretical basis and clinical tools for NB precision medicine.

### 3.1. Lactylation Is a Core Metabolic Feature of NB Tumor Cells

Lactylation is a newly discovered protein post-translational modification closely coupled with cellular glycolysis. Previous studies have confirmed its oncogenic roles in multiple malignancies, yet its distribution and biological function in neuroblastoma (NB) remain poorly defined. The present study annotated seven major cell types in NB tissues via scRNA-seq and distinguished tumor cells from normal neuroendocrine cells by CNV analysis, addressing the challenge of neuroendocrine cell heterogeneity in conventional cell annotation [23]. Lactylation activity was significantly upregulated in NB tumor cells, a finding mediated by the high expression of *LDHA* and *ENO1* in the high-lactylation group (avg_log2FC = 1.11 and 1.12, respectively). *ENO1* is a key glycolytic enzyme that catalyzes the conversion of 2-phosphoglycerate to phosphoenolpyruvate, driving glycolysis and promoting pyruvate accumulation [24]. *LDHA* acts as the core enzyme for lactate generation, specifically catalyzing the reduction of glycolysis-derived pyruvate to lactate [21]. The coordinated action of the two enzymes markedly increases lactate production in NB tumor cells, which aligns with the Warburg effect—a hallmark of cancer metabolic reprogramming characterized by enhanced glycolytic activity in both hypoxic and aerobic conditions [25]. Excess lactate accumulation provides sufficient substrate for lactylation modifications and further amplifies cellular lactylation activity, ultimately forming a vicious cycle of “glycolysis–lactate production–lactylation activation–tumor proliferation” [26]. Collectively, the single-cell evidence confirms that lactylation is a specific metabolic feature of NB tumor cells, and lays a theoretical foundation for targeting lactylation in NB treatment.

### 3.2. Lactylation May Be Associated with NB Progression via Regulating Chromosomal Stability and Cell Cycle

#### 3.2.1. Lactylation May Promote Chromosomal Instability by Disrupting Mitotic Checkpoints

Given the significant enrichment of DELGs in pathways governing chromosomal instability and cell-cycle dysregulation, we further investigated the relationship between lactylation and CIN, with particular attention to its association with cell-cycle progression.

##### The Sororin–Wapl–Cohesin Axis

Sororin (encoded by *CDCA5*) safeguards sister chromatid cohesion by antagonizing the cohesin-releasing factor Wapl during interphase and early mitosis [27,28]. To allow for timely separation in anaphase, the inhibition of Wapl must be transiently relieved, a process facilitated by the phosphorylation of Sororin by mitotic kinases such as *CDK1* and Aurora B [29,30]. In the present study, the high-lactylation NB cell population exhibited marked upregulation of *CDK1*, *AURKB*, and *CDCA5* (avg_log2FC = 3.37, 3.08, and 2.57, respectively). While direct evidence for lactylation modification of Sororin or its regulatory partners remains limited, recent studies demonstrate that lactylation can modulate mitotic kinase function. For instance, *TPX2* lactylation was identified as a critical regulator of AURKA kinase activity in hepatocellular carcinoma. Combined with the well-established role of *NBS1* and *MRE11* lactylation in maintaining genome stability through homologous recombination repair [31], our data support the hypothesis that elevated lactylation in NB may be correlated with disruptions in mitotic fidelity via lactylation-dependent protein modifications. We hypothesize that the concurrent overexpression of *CDK1*, *AURKB*, and *CDCA5* in high-lactylation cells could be associated with hyperphosphorylation of Sororin, potentially weakening its ability to inhibit Wapl. The consequent premature and widespread dissociation of cohesin, particularly at centromeric regions, may be linked to chromosome mis-segregation events, resulting in aneuploidy and fostering CIN—a known driver of tumor progression and therapy resistance [32].

##### The *PTTG1*–Securin–Separase Axis

Parallel to the cohesin pathway, the *PTTG1*–Securin–Separase axis serves as a critical gatekeeper of anaphase entry. *PTTG1* (pituitary tumor-transforming gene 1) is a known oncogene whose dysregulation contributes to aneuploidy [33,34]. It binds to and stabilizes Securin, which in turn directly inhibits separase to prevent premature sister chromatid separation before metaphase alignment [27,29]. In the high-lactylation group, a significant upregulation of *PTTG1* was observed (avg_log2FC = 2.80). Given that lactylation has been shown to regulate cell cycle protein stability and function, it is plausible that elevated lactylation may be correlated with altered *PTTG1* activity. The excess *PTTG1* could potentially disrupt the Securin–separase interaction, affecting Securin’s inhibitory function. The resultant premature activation of separase may be associated with untimely sister chromatid separation, further compounding CIN.

#### 3.2.2. Lactylation May Be Correlated with Accelerated Cell Cycle Progression

In addition to inducing genomic instability, the data indicated that lactylation directly propels the cell cycle machinery, expediting both the G1/S and G2/M transitions.

##### E2F1-Mediated G1/S Transition

As a key transcription factor governing the G1/S checkpoint, *E2F1* drives the expression of genes essential for DNA replication. Analysis revealed marked upregulation of *E2F1* in high-lactylation NB cells (avg_log2FC = 2.20). Histone lactylation has emerged as a direct epigenetic mechanism linking cellular metabolism to gene transcription. Notably, in lung adenocarcinoma, *RB1* lactylation at K900 was shown to disrupt the *RB1*-*E2F1* tumor-suppressive complex, leading to cell cycle dysregulation [34]. While direct evidence for lactylation-mediated *E2F1* transcriptional activation in NB remains to be characterized, the upregulation of *E2F1* in high-lactylation cells, combined with concurrent elevation of its downstream targets (*FEN1*, *MCM3*, *PCNA*, and *RFC3*), suggests a potential correlation between lactylation and accelerated G1/S transition. Moreover, recent studies demonstrated that histone H4K5 lactylation is increased in neuroblastoma and correlates with poor prognosis [35]. Importantly, lactate has been shown to activate the E2F pathway to promote cell motility by up-regulating microtubule-modulating genes, providing mechanistic clues to E2F-mediated transcriptional activation [36].

##### *CDK1*-Driven G2/M Transition

The G2/M transition is controlled by the activation of the Cyclin B-*CDK1* complex [37,38]. The Cyclin B-*CDK1* complex is kept inactive by inhibitory phosphorylation mediated by kinases such as Wee1 and Myt1 [39]. Once a threshold of active *CDK1* is reached, it creates a positive feedback loop with Cdc25B phosphatases [40,41]. In the present study, both *CDK1* and its activator *CDC25B* were significantly upregulated in the high-lactylation group (avg_log2FC = 3.37 and 2.21, respectively). Given that lactylation of *TPX2* was shown to enhance AURKA kinase activity and drive cell cycle progression, we hypothesize that lactylation may similarly be correlated with altered *CDK1* function. The observed upregulation may potentiate the Cdc25B-*CDK1*-positive feedback loop, leading to potential hyperactivation of *CDK1*. *CDK1* hyperactivation, in turn, may be associated with the premature activation of downstream mitotic kinases like Plk1 and phosphorylation of key substrates such as Rb, thereby correlating with accelerated mitotic entry and progression [42,43].

In sumctylation-related prognostic model.mary, our correlative analysis suggests that altered lactylation status is closely associated with two malignant phenotypes in NB: chromosomal instability and dysregulated cell cycle. Multiple published studies have confirmed that lactylation modulates mitotic kinases, DNA repair proteins, and histone functions across tumors. Nevertheless, this bioinformatics study cannot confirm causal relationships. Further lactylome profiling and functional experiments are required to verify the proposed regulatory mechanisms in neuroblastoma.

All differentially expressed lactylation-related genes (DELGs) above were included in the candidate pool for prognostic model construction. The close association between these cell cycle- and genome stability-related genes and tumor malignant phenotypes explains why lactylation-related genes possess reliable prognostic ability in NB, which also rationalizes the design of our 14-gene.

**Figure 9:**
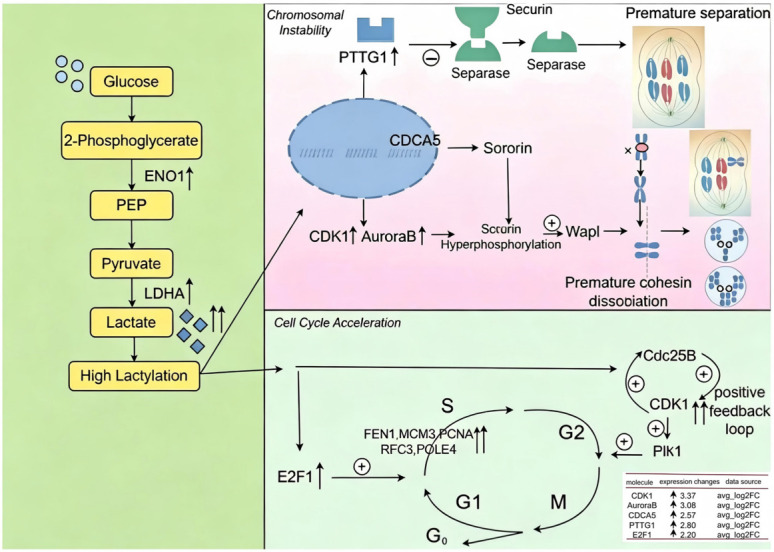
Lactylation is associated with neuroblastoma progression through potential links to chromosomal instability and cell-cycle dysregulation.

A schematic diagram summarizing the proposed mechanisms is presented in Figure 9.

### 3.3. Performance and Clinical Relevance of the Prognostic Model

#### 3.3.1. Superior Predictive Performance of the Lactylation-Related Prognostic Model

Currently, age at diagnosis, *MYCN* amplification, and INSS stage are the most widely used clinical prognostic indicators for NB, but their predictive efficacy is limited due to tumor high heterogeneity. In this study, we constructed a 14-gene lactylation-related prognostic model based on lactylation-related differentially expressed genes (DELGs) derived from scRNA-seq data. The model exhibited excellent discriminative power with a C-index of 0.901, and highly significant statistical results (*p* = 1.207 × 10^−48^). Rigorous internal and external cohort validation confirmed its stable predictive ability: the 3–5-year AUC values were all above 0.83 across three independent cohorts. Further multivariate Cox regression demonstrated that the risk score derived from our model was an independent prognostic factor for NB overall survival, independent of conventional clinical indicators. C-index, time-dependent ROC, and DCA analyses consistently proved that our model outperformed age, *MYCN* status, and INSS stage in risk discrimination and clinical net benefit. Compared with traditional markers and existing transcriptomic models for NB, this lactylation-related signature has higher accuracy and better generalizability.

Importantly, we benchmarked our model against recently published NB prognostic systems with comparable performance metrics. Our 14-gene lactylation-related risk score achieved a C-index of 0.901 in the training cohort and 0.818 in the external validation cohort, substantially outperforming the clinical nomogram based on SEER data (C-index: 0.790) [23] and the deep learning imaging model (mean C-index: 0.84) [8]. Even compared with the latest lactylation-related signature [22], which incorporated experimental validations and a 7-gene classifier, our model demonstrated a higher overall C-index (0.901 vs. ~0.84) and superior generalizability across three independent cohorts, despite the absence of wet-lab experiments in the current study. Notably, our 14-gene signature shares no overlapping genes with their 7-gene signature, suggesting that our model captures a distinct biological axis—cell cycle dysregulation and chromosomal instability—whereas theirs primarily reflects metabolic–immune crosstalk via the PI3K/AKT pathway. This comparison underscores that our purely transcriptomic, single-cell-derived model provides a unique, highly accurate, and clinically accessible tool for risk stratification, offering complementary value to existing imaging and clinical systems.

#### 3.3.2. Clinical Application Prospects

This 14-gene lactylation-related model has important practical value for NB clinical management. First, it can realize accurate risk stratification for NB patients: high-risk patients can receive intensified combined therapy, while low-risk patients can avoid overtreatment, achieving individualized treatment. Second, since the signature is closely linked to lactylation metabolism, it also provides clues for targeted therapy. Patients with high risk scores correspond to high lactylation activity, who may be more sensitive to lactylation or glycolysis inhibitors. In summary, the model acts as a dual functional tool for both prognostic evaluation and therapeutic guidance.

#### 3.3.3. Biological Rationality Verified by Single-Cell Expression Profiling

To exclude potential false positive results caused by bulk transcriptome heterogeneity, we further analyzed the single-cell expression landscape of the 14 signature genes. As revealed by violin plots and *t*-SNE maps (Figure 8), most risk genes (*ARC*, *CCT5*, *CENPN*, etc.) were specifically highly expressed in tumor cells, while protective genes (*TUBA1B*, *HIST1H4C*, *STAT3*, etc.) were predominantly enriched in tumor cells among all cell populations. This cell-type-specific expression pattern indicates that the prognostic efficacy of the signature is directly derived from the malignant characteristics of NB tumor cells, rather than infiltrating immune or stromal cells. This single-cell evidence strongly supports the biological reliability of our model and effectively makes up for the limitation of simple bulk data modeling.

### 3.4. Opportunities for Future Investigation

Several limitations of the present study need to be acknowledged. First, this study is a retrospective bioinformatics analysis based on publicly available datasets. Although our 14-gene lactylation-related prognostic model demonstrated robust prognostic performance in multiple independent cohorts and was further supported by single-cell mRNA expression analysis, future experimental validation at the mRNA and protein levels in independent prospective clinical samples would further strengthen and confirm its clinical applicability. Second, lactylation activity in this study was inferred from the expression of 371 lactylation-related genes, rather than being directly measured at the protein level. Future studies incorporating direct detection of lactylation modifications (e.g., by Western blot with a pan-lactyl-lysine antibody or mass spectrometry-based lactylome profiling) are needed to validate our findings. Third, the current model only incorporates lactylation-related genes; combining our model with classic clinical indicators (*MYCN*, INSS stage) may further improve predictive performance.

For future research, multiple directions can be carried out. Clinically, we will collect multi-center prospective NB samples to complete qRT-PCR and immunohistochemistry validation for the 14 core genes and optimize the model combined with clinical parameters. Mechanistically, in vitro cell and in vivo animal experiments are needed to clarify the regulatory relationship between key signature genes and lactylation modification, and to explore the anti-tumor effect of lactylation inhibitors in high-risk NB cells. Collectively, these follow-up works will promote the clinical transformation of our findings.

## 4. Materials and Methods

### 4.1. Data Acquisition

ScRNA-seq data of NB (GSE137804, 4 cases) were downloaded from the Gene Expression Omnibus (GEO, https://www.ncbi.nlm.nih.gov/geo/ (accessed on 25 October 2025)) database [44], for single-cell transcriptomic profiling, including cell clustering, annotation, and lactylation activity analysis. To quantify lactylation activity at the single-cell level, a total of 371 lactylation-related genes (LRGs) (Table S3) were obtained from a published study, which compiled the gene set through a systematic review of previous studies [45]. This gene set encompasses multiple functional categories within the lactylation modification pathway, including lactylases (e.g., *EP300*), genes encoding proteins experimentally identified as lactylation substrates (e.g., the histone *H4C1* and the metabolic enzyme *LDHA*), and other lactylation-related genes reported in the literature. The gene set has been previously validated for lactylation activity assessment in tumor cells. The established gene panel was therefore adopted for lactylation activity calculation in the present scRNA-seq analysis. For bulk RNA-seq data of neuroblastoma patients, the GEO dataset GSE49710 (498 cases) was randomly split into a training cohort (*n* = 349) and an internal validation cohort (*n* = 149) [43]. The ArrayExpress (https://www.ebi.ac.uk/biostudies/arrayexpress/studies/ (accessed on 13 November 2025)) dataset E-MTAB-8248 containing 223 neuroblastoma patients served as the independent external validation cohort [44].

### 4.2. Data Processing and Cell Annotation for scRNA-Seq

ScRNA-seq data were processed using the ‘Seurat’ R package (version 5.3.1) with standard quality control (QC): cells with 200 ≤ genes ≤ 5000, 200 ≤ unique molecular identifiers (UMIs) ≤ 30,000, mitochondrial gene ratio ≤ 20%, and hemoglobin gene ratio ≤ 5% were retained. The data were normalized by the LogNormalize method, and 2000 highly variable genes (HVGs) were selected via the vst method. PCA was performed on HVGs, and the top 10 principal components were used for K-nearest neighbor clustering and Louvain algorithm-based cell clustering (resolution = 1). Cell types were annotated by marker gene expression patterns via the DotPlot function. CNV analysis by CopyKAT R package (version 1.1.0) was used to distinguish aneuploid tumor cells from diploid normal neuroendocrine cells, with myeloid cells, T cells, pDCs and B cells as diploid references.

### 4.3. Lactylation Activity Scoring and Identification of DELGs

Lactylation activity scores were first computed for individual cells using the AddModuleScore framework with 371 predefined LRGs. The median score served as the stratification boundary to classify cells into high- and low-lactylation groups. To identify genes associated with lactylation status, differential expression analysis was performed comparing the two tumor cell subsets via Wilcoxon rank-sum testing. Candidate DELGs were selected based on multiple criteria: min.pct ≥ 0.25, logfc.threshold ≥ 0.25, adjusted *p <* 0.05, target_pct_threshold ≥ 0.25, and |avg_log2FC| > 1. The candidate DELGs were finally represented through volcano plot visualization. The multi-step process generated the final set of differentially expressed lactylation-related genes (DELGs), which were visualized in a volcano plot.

### 4.4. Functional Enrichment Analysis and PPI Network Construction

Gene Ontology (GO) and Kyoto Encyclopedia of Genes and Genomes (KEGG) pathway enrichment analyses of DELGs were performed using the ‘clusterProfiler’ R package (version 4.18.2) (adjusted *p <* 0.05), and the results were visualized by the ‘*ggplot2*’ package. The protein–protein interaction (PPI) network of DELGs was constructed via the STRING database (confidence score ≥ 0.7) and imported into Cytoscape for visualization. The CytoNCA plugin was used to calculate betweenness centrality (BC) of each node, and genes in the top quartile of BC values were defined as core genes.

### 4.5. Prognostic Model Construction and Validation

#### 4.5.1. Candidate Gene Selection and Data Preprocessing

Candidate genes were derived from single-cell RNA-seq (scRNA-seq) data of neuroblastoma (GSE137804). Differentially expressed lactylation-related genes (DELGs, *n* = 142) were identified by comparing high- versus low-lactylation tumor cells and served as the gene pool for prognostic model development. The expression levels of the DELGs were then extracted from two bulk RNA-seq cohorts: GSE49710 (*n* = 498) for model construction and internal validation, and E-MTAB-8248 (*n* = 223) for external validation.

The GSE49710 bulk RNA-seq cohort was randomly divided into a training set (70%, *n* = 349) and an internal validation set (30%, *n* = 149) using stratified sampling based on the outcome event (fustat). A fixed random number seed of 123 was set to enable reproducible sample partitioning. Baseline characteristics (age, sex, *MYCN* amplification status, INSS stage, and overall survival) were compared between the two sets, and no significant differences were observed (all *p >* 0.05). The E-MTAB-8248 cohort was used as an independent external validation set without further partitioning.

#### 4.5.2. Construction of the 14-Gene Lactylation-Related Prognostic Model

The prognostic risk score model was constructed based on the following steps:

Step 1. Univariate Cox regression: Genes significantly associated with overall survival (OS) were identified using univariate Cox proportional hazards regression analysis. Genes with *p <* 0.05 were retained as candidate prognostic genes.

Step 2. LASSO regression: The candidate genes were further processed by the least absolute shrinkage and selection operator (LASSO) Cox regression using the R package glmnet with 10-fold cross-validation and a fixed random seed (seed = 2) to ensure reproducibility. The optimal λ value (lambda.min) was selected, yielding 20 genes with non-zero coefficients.

Step 3. Multivariate Cox regression and stepwise selection: The genes selected by LASSO were included in a multivariate Cox regression model, and bidirectional stepwise selection was applied using the R step function with direction = “both” and a threshold of *p* = 0.05 for variable entry and retention. The most parsimonious set of genes with independent prognostic value was identified.

Step 4. Risk score calculation: Gene expression levels were log_2_(x + 1)-transformed prior to risk score calculation. The risk score for each patient was calculated using the linear combination of the expression levels of the final selected genes weighted by their regression coefficients from the multivariate

$$\mathrm{Cox}\;\mathrm{model}:Risk\;Score=\sum_{i=1}^{n}\left(\beta_{i}\times {Gene}_{i}\right)$$

where:

$\beta_{i}$ = the regression coefficient of the corresponding gene in the multivariate Cox model;

${Gene}_{i}$ = the expression level of each gene after $\log_{2}\left(x+1\right)$ transformation; 

***n*** = the total number of independent prognostic genes incorporated into the model.

Step 5. Risk stratification: The optimal cut-off value for the risk score was determined using the ‘surv_cutpoint’ function in the ‘survminer’ (version 0.5.2) package based on the Log-rank test in the training cohort, and patients were stratified into high- and low-risk groups accordingly.

#### 4.5.3. Model Evaluation and Validation

The performance of the lactylation-related gene risk model was evaluated in the training set, internal validation set (30% of GSE49710 samples), and external validation set (E-MTAB-8248).

Evaluation metrics and methods:

① Time-dependent ROC curves: The ‘timeROC’ R package was used to calculate the area under the curve (AUC) for 3-year, 4-year, and 5-year overall survival.
② Kaplan–Meier survival curves and log-rank test: Survival curves for the high- and low-risk groups were plotted, and the difference in overall survival between the two groups was compared using the two-sided log-rank test.
③ Risk distribution plot: Generated using the R package ‘*ggplot2*’ to display the distribution of risk scores and survival status of patients.
④ Heatmap of model gene expression: Showing the expression differences of the 14 model genes between the high- and low-risk groups.
⑤ Overall model performance assessment: The concordance index (C-index), global likelihood-ratio test (*χ^2^* and *p* value), and Akaike Information Criterion (AIC) were calculated.

#### 4.5.4. Comparison of the Risk Score Model with Traditional Clinical Prognostic Indicators

Multivariate Cox proportional hazards regression was performed to assess the independent prognostic value of the 14-gene risk score and conventional clinical indicators in the external validation cohort (E-MTAB-8248). All clinical variables were dichotomized according to established clinical criteria: age (≥1.5 vs. <1.5 years), *MYCN* amplification (amplified vs. non-amplified), and INSS stage (stage 4/4S vs. stage 1–3). The concordance index (C-index) was calculated to compare the discriminative ability between the risk score model and traditional factors. Time-dependent receiver operating characteristic (ROC) curves were constructed to evaluate the predictive performance of the risk score and clinical indicators for 1-, 3-, and 5-year overall survival (OS). Decision curve analysis (DCA) was applied to compare the clinical net benefit of different models. A two-sided *p* < 0.05 was considered statistically significant.

### 4.6. Single-Cell Expression Landscape of the 14-Gene Lactylation-Related Prognostic Model in Neuroblastoma

To clarify the cell-specific expression patterns of the prognostic model’s core genes, we utilized the previously annotated scRNA-seq data. This dataset encompassed seven major cell types, including tumor cells, normal neuroendocrine cells, and fibroblasts. Expression levels of the 14 model genes across these different cell types were visualized using the ‘VlnPlot’ function from the ‘Seurat’ R package. The width of each violin plot reflects the cell density at corresponding expression levels. The ‘FeaturePlot’ function was employed to generate *t*-SNE spatial maps, which illustrate the distribution of gene expression within cell clusters using a color gradient to represent expression intensity. These plots provided a direct visualization of expression features. By integrating each gene’s prognostic role (as a risk or protective factor) with its cellular expression localization, we analyzed the intrinsic links between gene expression patterns and neuroblastoma prognosis.

## 5. Conclusions

The present study integrated single-cell and bulk transcriptome data to systematically explore the role of lactylation modification in neuroblastoma (NB). At the single-cell level, we verified that NB tumor cells exhibit a unique high-lactylation metabolic phenotype. A total of 142 lactylation-related differentially expressed genes (DELGs) were screened out by comparing high- and low-lactylation tumor populations. Functional enrichment analyses indicated that these DELGs are primarily involved in cell cycle and DNA replication pathways. Mechanistically, correlative analyses suggest that altered lactylation status may participate in NB malignant phenotypes through two potential regulatory axes: it is potentially associated with chromosomal instability caused by disrupted mitotic checkpoints, as well as dysregulated cell cycle progression.

Based on the identified DELGs, we established a 14-gene lactylation-related prognostic model via univariate Cox regression, LASSO regression, and Multivariate Stepwise Cox regression. The model yielded a C-index of 0.901 and exhibited outstanding predictive capacity, which was validated in training, internal, and external independent cohorts. Time-dependent ROC curves, Kaplan–Meier survival analysis, and decision curve analysis (DCA) confirmed that this signature acts as an independent prognostic indicator. It shows higher predictive accuracy and clinical net benefit than conventional clinical markers, including age, *MYCN* amplification, and INSS stage. Furthermore, single-cell expression profiling verified that the 14 signature genes are predominantly expressed in NB tumor cells, further supporting the biological rationality of the established model.

This study elucidates the potential association between lactylation and the malignant characteristics of NB, and provides a novel, reliable molecular tool for patient risk stratification and individualized treatment. Meanwhile, lactylation is highlighted as a promising therapeutic target for NB. Considering that this is a retrospective bioinformatics study, additional prospective clinical validation and mechanistic functional experiments are still required to consolidate our findings and advance their clinical translation. This work also offers new insights for the precision diagnosis and targeted therapy of neuroblastoma.

## Data Availability

The single-cell RNA sequencing data analyzed in this study are available in the Gene Expression Omnibus (GEO) database under accession number GSE137804. The training and internal validation cohorts were derived from the GEO dataset GSE49710 (https://www.ncbi.nlm.nih.gov/geo/ (accessed on 12 May 2026)). The external validation cohort was obtained from the ArrayExpress database under study accession E-MTAB-8248 (https://www.ebi.ac.uk/biostudies/arrayexpress/studies/ (accessed on 12 May 2026)). The lactylation-related gene set (LRGs) is available in Table S3.

## Supplementary Materials

The following supporting information can be downloaded at: https://www.mdpi.com/article/10.3390/molecules31132280/s1, Table S1: Differentially expressed lactylation-related genes (DELGs); Table S2: Betweenness centrality calculation results of lactylation-related DELGs in the PPI network (via CytoNCA plugin); Table S3: Lactylation-Related Genes (LRGs).
